# The effect of balneotherapy and peloid therapy on changes in the functional state of patients with knee joint osteoarthritis: a randomized, controlled, single-blind pilot study

**DOI:** 10.1007/s00484-019-01785-z

**Published:** 2019-09-06

**Authors:** Lina Varzaityte, Raimondas Kubilius, Lolita Rapoliene, Ruta Bartuseviciute, Arvydas Balcius, Kestutis Ramanauskas, Irena Nedzelskiene

**Affiliations:** 1 Department of Rehabilitation, Lithuanian University of Health Sciences, A. Mickevičiaus str. 9, LT-44307 Kaunas, Lithuania; 2 Department of Nursing, Klaipeda University, H. Mantas str. 84, LT-92294 Klaipėda, Lithuania; 3 Limited company Medical SPA Eglės Sanatorija, Eglės str. 1, LT-66251 Druskininkai, Lithuania; 4 Druskininkai Recreation and Health Centre, Vilniaus av. 11, LT-66119 Druskininkai, Lithuania; 5 Department of Dental and Oral Pathology, Lithuanian University of Health Sciences, Mickevičiaus str. 9, LT-44307 Kaunas, Lithuania

**Keywords:** Knee joint osteoarthritis, Balneotherapy, Peloid therapy, Rehabilitation

## Abstract

The treatment of OA using pharmaceutical and non-pharmaceutical measures remains a topical subject. The purpose of this study is to assess the effect of natural factors (mineral water and mud) on changes in the functional state of patients with knee joint OA. Ninety-two adult people with grade I–III knee joint OA according to the Kellgren and Lawrence scoring system participated in the study. The subjects received 10 mineral water bath plus physical therapy or mud application procedures plus physical therapy or physical therapy alone every other day. The effectiveness of the treatment was assessed on the basis of anthropometric changes of data, VAS, SF-36, KOOS questionnaire indicators. Significantly greater walking speed, test of 5 sit downs/stand ups, circumference of a knee joint, flexion and extension range, flexor and extensor strength after treatment lasting 1 month were obtained in the intervention group. After 1 month after treatment pain intensity scores over the past month and when changing position were significantly higher in the control group. The positive changes in SF-36 were identified after 1 month after treatment: physical activity increased and pain decreased in the intervention groups. There was no significant difference between the averages of any KOOS subscale in groups. However, average percentages of symptoms, stiffness, and pain in the intervention groups were significantly better after treatment and lasting 1 month after treatment. Balneotherapy and peloid therapy effectively reduce pain and improve the functional state of patients with OA of a knee joint.

## Introduction

Knee joint osteoarthritis (OA) is a chronic degenerative knee joint disease, characterized by anatomical and/or physiological disorders, which manifest in the degeneration of the joint cartilage, bone tissue rearrangement, formation of osteophytes, synovial membrane inflammation, joint capsule, and ligament damage as well as loss of normal function (Kraus et al. 2015). These processes lead to clinical symptoms of the disease—dull pain of aching nature or sharp, intermittent knee joint pain, crepitation, swelling as well as stiffness, decrease of movement amplitude, and leg muscle weakness (Anandkumar et al. 2014; Campbell et al. 2015). According to data from the World Health Organization (2013), in total, 9.6% of men and 18.0% of women older than 60 years of age suffer from symptomatic OA. Combination therapy is recommended for treatment of OA, using pharmaceutical and non-pharmaceutical measures at the same time, the purpose of which is to relieve pain, to slow down the progression of the disease and to improve or compensate for impaired movement function (Bruyère et al. 2014). The most commonly recommended non-pharmaceutical treatment measures are as follows: physiotherapy, weight correction, orthopedic or technical measures, electrical stimulation or treatment with other physical agents (Cutolo et al. 2015; McAlindon et al. 2014). Natural factors (balneotherapy or peloid therapy) have been referred to more and more in literature as treatment for musculoskeletal diseases (Karagülle and Karagülle 2015, Verhagen et al. 2015).

Balneotherapy traditionally means bathing in mineral and/or thermal water from natural sources. On the other hand, treatment with gas molecules (e.g. CO2, H_2_S), mud or other natural factors in literature is often attributed to balneotherapy interventions (Fioravanti et al. 2017).

The aim of this study was to assess the effect of natural factors—mineral water and mud—on changes in the functional state of patients with knee joint OA.

## Materials and methods

Clinical parameters (walking speed, time of 5 sit downs/stand ups, circumferences of thigh, knee, and calf, flexion/extension ranges, flexor/extensor strength) were assessed of all subjects during stage I (before treatment), stage II (after treatment), and stage III (after 1 month after treatment). Subjects’ pain was assessed using visual analogue pain scale (VAS), where pain intensity is represented by a point between 0 and 10 (from “no pain” to “unbearable pain”). Subjects were assessed using life quality assessment questionnaire (SF-36v2® Health Survey Standard, Lithuania (Lithuanian)). It consists of 36 questions, which reflect eight sections of life: physical activity, restriction of activity due to physical problems, pain, overall health assessment, energy levels and vitality, social function, restriction of activity due to emotional disorders as well as emotional state. Answers to these questions are scored. Each section is scored from 0 to 100 using calculation algorithm. The higher the score, the better the quality of life. Distribution of validity of the scales of SF-36 questionnaire sections (Cronbach’s alpha coefficient) at different stages of the study was as follows: at stage I—0.745, at stage II—0.764, at stage III—0.732 (The stability of the questionnaire over time is considered to be good enough when the interclass correlation coefficient is equal or greater than 0.70). Subjects were assessed using Knee injury and Osteoarthritis Outcome Score (KOOS) that is validated and adapted in Lithuania (Mapi research institute 2007). It is a subjective assessment method of functional state and quality of life in relation to the knee joint. It consists of 5 subscales—symptoms, stiffness, pain, mobility, everyday life and mobility, sports and recreational activities. The questionnaire was used for the assessment of respondents of all subjects during stage I (before treatment), stage II (after treatment), and stage III (after 1 month after treatment). On the basis of assessment methodology, presented on the official webpage of the questionnaire, assessments of each scale within a 100% system were obtained, where 100 means absence of symptoms, while 0 means significantly expressed symptoms. Validity of the questionnaire scale was also assessed. It was found that Cronbach’s alpha of stage I was 0.959, stage II—0.975, and stage III—0.977, i.e., strong internal scale consistency was demonstrated.

Statistical analysis was performed using Excel and SPSS 22. Descriptive statistical characteristics of data were calculated as follows: for quantitative data − the average and its standard deviation. Compliance of distribution of quantitative variables to normal distribution was tested using the Kolmogorov–Smirnov test.

The means of three groups were compared using the dispersion analysis (ANOVA). For multiple comparisons, Bonferroni test (post hoc) was applied. When the test of normality of the investigated variables was denied, Kruskal–Wallis and the Mann–Whitney tests were used (*F*—Fisher test, χ^2^—Kruskal–Wallis test). Between two dependent groups, non-parametric Wilcoxon signed ranks criteria were used. Interdependence of categorical variables was assessed by employing chi-square criteria. ROC (receiver operating characteristic) curve analysis method was used for determination of optimal values of parameters. The optimal values separated out the different groups with the highest accuracy. The logistic regression analysis was performed to determine odds ratio of participants with intervention group. Difference or relationship was considered to be statistically significant when the significance of the criteria was *p* < 0.05.

For a probability to reasonably reject a false hypothesis, we calculated power of the study (when type 1 error *α* ≤ 0.05).

Power calculations were based on the article by Branco et al. (2016) “Bath thermal waters in the treatment of osteoarthritis of the knee: a randomized controlled clinical trial”.

The results of the final study proved that H0 hypothesis was reasonably rejected, obtaining powers of the study > 0.9 (pain on movement: baseline—6.9 (2.1), after treatment—4.5 (2.5); pain at night: baseline—4.2 (2.9), after treatment—2.4 (2.2); pain at rest: baseline—3.4 (2.4); after treatment—1.7 (1.7).

## Description of the research contingent

**Fig. 1:**
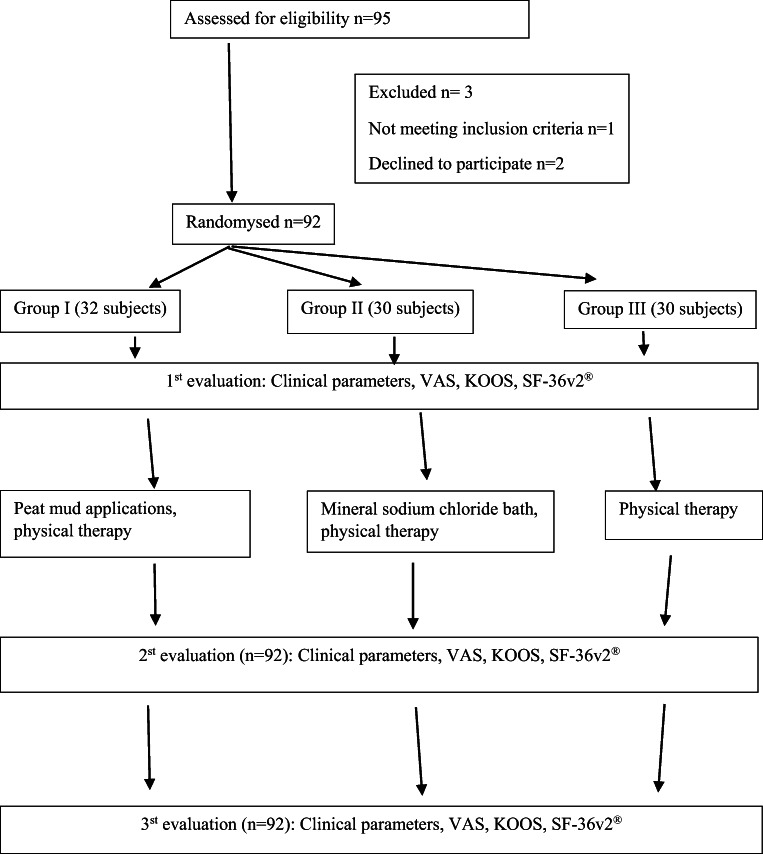
Trial organization scheme

This study was a randomized, controlled, single-blind comparison between balneotherapy plus physical therapy, mud therapy plus physical therapy, and physical therapy alone, which took place from 29/06/2018 to 31/12/2018, after obtaining Kaunas Regional Biomedical Research Ethics Committee permission (Nr. BE-2-65). All subjects signed the informed consent form prior to the initiation of the study. The inclusion criteria were as follows: subjects 18 years of age and older, with grade I–III knee joint OA according to the Kellgren and Lawrence scoring system participated in the study. Exclusion criteria were endoprosthetic knee and hip joints, rheumatoid arthritis, podagra, systemic connective tissue disease, failure to perform functional tests for the study due to significant knee joint disorder or related pathology, sensitive or damaged skin in the area of dirt and/or mineral water, oncologic disease diagnosed or suspected, fewer than 6 months after intraarticular injections, permanent use of pain medication due to comorbidity, pregnancy, pregnancy planning in the near future, refusal, participation in the research, no use of healing lubricants, knee joint, and misunderstanding of the Lithuanian language. The trial involved 95 adults with grade I–III knee joint OA. The subjects were randomly assigned to study groups with a 1:1:1 allocation ratio, according to the rehabilitation registration journal. A total of 92 subjects established the trial, and 3 subjects left the trial because of the following reasons: 1 not meeting inclusion criteria, and 2 subjects refused to participate in the trial (Fig. 1).

Participants of group I (32 subjects) were treated using peat mud applications in the waist and leg area, 36–42 °C temperature, duration of the procedure 20 min, 10 procedures every other day, specific physical therapy (10 procedures every other day) at sanatorium “Egle,” in Druskininkai. Participants of group II (30 subjects) were treated using the mineral sodium chloride bath with mineralization 40–46 g/l, temperature of water 36–38 °C, duration of the procedure 15 min, 10 procedures every other day, specific physical therapy (10 procedures every other day) at sanatorium “Egle,” in Druskininkai. Participants of group III (30 subjects), the control group, received specific physiotherapy (10 procedures every other day) at the Hospital of the Lithuanian University of Health Sciences, Rehabilitation Clinic. The physical therapy was standardized in all three groups. All physical therapists had the same protocol with exercises, which was discussed before the study. The subjects received the same exercises, for the same repetition and number of sets, to increase the amplitude of motion of the knee joint, for muscle strengthening and proprioception training. The specific physiotherapy program consisted of 10 procedures every other day and the duration of the procedure was 30 min.

Physical therapy program consisted of warm up exercises for 5 min—to improve blood circulation and activate the muscles for the main part. Main part (20 min) physical therapy exercises were made to maintain and improve joint function, mobility, and flexibility. Strengthening exercises were performed in a closed and opened kinematic chain for quadriceps and hamstrings, followed by exercises to strengthen the stabilizing muscles of knee, hip, and ankle joint. Proprioception and gait training were also performed in the main part. Muscle stretching exercises were performed in a cooling down (5 min) part.

The mineral sodium chloride bath, peat mud applications, and physiotherapy were safe and the subjects tolerated them well. In general, the side effect was not observed. The assessors were blinded to the research condition by not being able to check the patient status.

## Results

The study presents the results of the 92-subject trial. The mean age of the subjects was 64.6 (11.4) years. The majority of the subjects was females—87.0%, males constituted 13.0%. The mean body weight of subjects was 29.4 (4.3). The baseline characteristics of subjects (age, sex, body mass index), with respect to groups, did not differ significantly (Table 1).

**Table 1:** The baseline characteristics of subjects (age, sex, body mass index), with respect to groups of subjects

Characteristic	All groups (*n* = 92)	Group I (*n* = 32)	Group II (*n* = 30)	Group III (*n* = 30)	
Age, A (SD)	64.6 (11.4)	65.0 (10.8)	61.0 (13.4)	67.9 (8.9)	*p* = 0.062
Sex (%)					*χ*^2^ = 3.521 *p* = 0.172
Men	13.0	21.9	10.0	6.7	
Women	87.0	78.1	90.0	93.3	
Body mass index, A (SD) kg/m^2^	29.4 (4.3)	29.3 (3.9)	29.2 (4.6)	29.8 (4.6)	*p* = 0.873

Group I, provided with bath procedures; group II, provided with mud procedures; group III, control; *A*, average; *SD*, standard deviation; *χ*^2^, chi-square criteria; *p*, level of significance.

After treatment significantly greater change in the intervention group (group I and group II) was in walking speed, test of 5 sit downs/stand ups flexion range, extension range of the knee of left leg, and flexor and extensor strength of left and right leg muscles, except circumference of right thigh and knee, circumference of left and right calf compared with the control group (Table 2).

**Table 2:** Distribution of change in clinical parameters after treatment (stages I–II), with respect to groups of subjects

Variable	Group I (*n* = 32)	Group II (*n* = 30)	Group III (*n* = 30)	*p* value
Walking speed, A (SD), m/s	0.20 (0.25)^a^	0.19 (0.28)^b^	− 0.24 (0.24)^a,b^	*F* = 27.984, ^a,b^*p* < 0.001
5 sit downs/stand ups, A (SD), s	3.83 (3.02)^a^	4.24 (3.79)^b^	1.98 (2.00)^a,b^	*χ*^2^ = 11.938, ^a^*p* = 0.005, ^b^*p* = 0.002
Left leg				
Thigh circumference, A (SD), cm	0.78 (1.38)	0.50 (1.46)	0.76 (1.73)	*χ*^2^ = 0.921, *p* = 0.631
Knee circumference, A (SD), cm	1.42 (2.80)	1.40 (2.22)	0.96 (1.94)	*χ*^2^ = 1.281, *p* = 0.527
Calf circumference, A (SD), cm	1.16 (1.17)	1.15 (1.29)	0.66 (1.50)	*F* = 1.264, *p* = 0.288
Flexion range, A (SD), degrees	16.2 (9.3)^a^	16.8 (14.3)^b^	5.4 (5.1)^a,b^	*χ*^2^ = 23.02, ^a,b^*p* < 0.001
Extension range, A (SD), degrees	1.92 (3.62)	1.0 (2.38)^b^	1.87 (2.17)^b^	*χ*^2^ = 6.309, ^b^*p* = 0.017
Flexor strength, A (SD), score	1.12 (0.44)^a^	1.04 (0.81)^b^	0^a,b^	*χ*^2^ = 51.417, ^a,b^*p* < 0.001
Extensor strength, A (SD), score	1.20 (0.76)^a^	1.25 (0.85)^b^	0.07 (0.25)^a,b^	*χ*^2^ = 42.192, ^a,b^*p* < 0.001
Right leg				
Thigh circumference, A (SD), cm	1.23 (1.75)	1.28 (1.80)	0.73 (2.16)	*χ*^2^ = 7.309, *p* = 0.503
Knee circumference, A (SD), cm	1.24 (1.40)^a^	1.12 (0.98)^b^	0.18 (1.58)^a,b^	*χ*^2^ = 8.807, ^a^*p* = 0.018,^b^*p* = 0.008
Calf circumference, A (SD), cm	1.22 (2.51)	1.10 (1.1)	0.35 (1.29)	*χ*^2^ = 7.348, *p* = 0.132
Flexion range, A (SD), degrees	19.0 (13.3)^a^	17.2 (12.9)^b^	5.10 (6.40)^a,b^	*χ*^2^ = 24.127, ^a,b^*p* < 0.001
Extension range, A (SD), degrees	2.12 (7.14)	1.88 (3.79)	2.0 (2.33)	*χ*^2^ = 2.422, *p* = 0.298
Flexor strength, A (SD), score	1.17 (0.48)^a^	1.08 (0.40)^b^	0^a,b^	*χ*^2^ = 62.551, ^a,b^*p* < 0.001
Extensor strength, A (SD), score	1.00 (0.80)^a^	1.13 (0.63)^b^	0.07 (0.25)^a,b^	*χ*^2^ = 41.327, ^a,b^*p* < 0.001

Group I, provided with bath procedures; group II, provided with mud procedures; group III, control; *A*, average; *SD*, standard deviation; *F*, by Fisher test; *χ*^2^, by Kruskal–Wallis test

^a,b^*p* < 0.05

After 1 month after treatment, all changes of the intervention group (group I and group II) were significantly better, except thigh, knee and calf circumference of the left leg, thigh, and calf circumference left leg, extension range of the left and right leg compared with the control group (Table 3).

**Table 3:** Distribution of change of subjects’ clinical parameters after 1 month after treatment (stages I–III), with respect to groups of subjects

Variable	Group I (*n* = 32)	Group II (*n* = 30)	Group III (*n* = 30)	*p* value
Walking speed, A (SD), m/s	0.28 (0.27)^a^	0.24 (0.25)^b^	− 0.20 (0.18)^a,b^	*F* = 37.846, ^a,b^*p* < 0.001
5 sit downs/stand ups, A (SD), s	5.40 (3.41)^a^	5.61 (3.72)^b^	2.10 (2.61)^a,b^	*χ*^2^ = 22.603, ^a,b^*p* < 0.001
Left leg				
Thigh circumference, A (SD), cm	1.38 (2.09)	1.02 (1.89)	0.78 (1.77)	*χ*^2^ = 2.082, *p* = 0.353
Knee circumference, A (SD), cm	2.0 (2.33)	1.67 (2.53)	1.11 (1.58)	*χ*^2^ = 2.835, *p* = 0.242
Calf circumference, A (SD), cm	1.14 (1.50)	1.87 (2.50)	0.74 (1.55)	*χ*^2^ = 5.167, *p* = 0.076
Flexion range, A (SD) degrees	19.9 (10.8)^a^	18.8 (17.5)^b^	4.20 (5.50)^a,b^	*χ*^2^ = 31.944, ^a,b^*p* < 0.001
Extension range, A (SD), degrees	2.36 (4.56)	0.83 (2.50)	1.77 (2.22)	*χ*^2^ = 5.393, *p* = 0.067
Flexor strength, A (SD), score	1.28 (0.54)^a^	1.22 (0.85)^b^	0^a,b^	*χ*^2^ = 49.015, ^a,b^*p* < 0.001
Extensor strength, A (SD), score	1.36 (0.91)^a^	1.30 (0.97)^b^	0.07 (0.25)^a,b^	χ^2^ = 39.506, ^a,b^*p* < 0.001
Right leg				
Thigh circumference, A (SD), cm	1.67 (1.96)	1.69 (2.09)	0.91 (2.13)	*F* = 1.282, *p* = 0.283
Knee circumference, A (SD), cm	1.80 (1.65)^a^	1.52 (1.53)^b^	0.33 (1.74)^a,b^	*χ*^2^ = 13.476, ^a^*p* = 0.001,^b^*p* = 0.004
Calf circumference, A (SD), cm	1.34 (2.29)	1.50 (1.81)	0.36 (1.20)	*χ*^2^ = 5.658, *p* = 0.059
Flexion range, A (SD), degrees	22.6 (15.9)^a^	18.8 (15.4)^b^	1.13 (8.89)^a,b^	*χ*^2^ = 32.642, ^a,b^*p* < 0.001
Extension range, A (SD), degrees	2.28 (7.59)	2.33 (4.46)	1.87 (2.97)	*χ*^2^ = 0.5, *p* = 0.779
Flexor strength, A (SD), score	1.24 (0.72)^a^	1.13 (0.54)^b^	0^a,b^	*χ*^2^ = 52.397, ^a,b^*p* < 0.001
Extensor strength, A (SD), score	1.20 (0.96)^a^	1.23 (0.92)^b^	0.07 (0.25)^a,b^	*χ*^2^ = 35.259, ^a,b^*p* < 0.001

Group I, provided with bath procedures; group II, provided with mud procedures; group III, control; *A*, average; *SD*, standard deviation; *F*, by Fisher test; *χ*^2^, by Kruskal–Wallis test

^a,b^*p* < 0.05

Based on VAS, after 1 month after treatment (stage III), pain intensity scores over the past month and when changing position were significantly higher in the control group compared with group I (Table 4).

**Table 4:** Distribution of the scores of subjects’ pain intensity questionnaire before, after treatment, and after 1 month after treatment (I, II, III stage), with respect to groups of subjects

	Group I (*n* = 32)	Group II (*n* = 30)	Group III (*n* = 30)	*p* value
	A (SD), score			
Over the past month				
Stage I	5.88 (1.66)	6.33 (2.34)	5.67 (2.51)	*F* = 0.729, *p* = 0.485
Stage II	4.44 (1.93)	4.57 (2.47)	5.27 (2.75)	*χ*^2^ = 2.774, *p* = 0.25
Stage III	3.63 (2.04)^a^	4.38 (2.44)	5.13 (2.66)^a^	*F* = 3.09, ^a^*p* = 0.044
During daytime				
Stage I	5.97 (1.91)	6.27 (2.10)	4.93 (2.52)	*χ*^2^ = 5.024, *p* = 0.081
Stage II	4.03 (2.10)	4.40 (2.34)	4.57 (2.74)	*F* = 0.406, *p* = 0.668
Stage III	3.25 (2.08)	4.48 (2.61)	4.57 (2.64)	*χ*^2^ = 4.934, *p* = 0.085
During night time				
Stage I	4.16 (2.60)	4.37 (3.18)	3.37 (3.58)	*χ*^2^ = 2.939, *p* = 0.23
Stage II	2.81 (2.16)	3.07 (2.57)	3.13 (3.69)	*χ*^2^ = 0.953, *p* = 0.621
Stage III	2.34 (1.98)	2.55 (2.38)	3.0 (3.73)	*χ*^2^ = 0.6, *p* = 0.741
When moving				
Stage I	6.69 (2.07)	7.17 (2.10)	5.73 (2.48)	*χ*^2^ = 5.553, *p* = 0.062
Stage II	4.81 (2.15)	5.33 (2.59)	5.40 (2.63)	*χ*^2^ = 1.172, *p* = 0.556
Stage III	4.25 (2.53)	4.72 (2.52)	5.20 (2.46)	*χ*^2^ = 2.194, *p* = 0.334
When changing position				
Stage I	6.69 (2.49)	6.90 (2.35)	6.03 (2.48)	*χ*^2^ = 2.371, *p* = 0.306
Stage II	4.66 (2.07)	5.0 (2.69)	5.90 (2.85)	*F* = 1.948, *p* = 0.149
Stage III	4.06 (2.50)^a^	4.55 (2.60)	5.77 (2.89)^a^	*F* = 3.325, *p* = 0.485, ^a^*p* = 0.047
At rest				
Stage I	3.47 (2.36)	3.40 (2.43)	3.03 (3.10)	*χ*^2^ = 1.299, *p* = 0.522
Stage II	2.28 (2.28)	2.77 (2.34)	2.87 (3.32)	*χ*^2^ = 0.967, *p* = 0.617
Stage III	1.50 (1.74)	1.83 (1.58)	2.93 (3.27)	*χ*^2^ = 2.319, *p* = 0.314

Group I, provided with bath procedures; group II, provided with mud procedures; group III, control; *A*, average; *SD*, standard deviation; *F*, by Fisher test; *χ*^2^, by Kruskal–Wallis test.

^a,b^*p* < 0.05

Analysis of separate areas of SF-36 questionnaire shows that the positive changes were identified after treatment and 1 month after the treatment in all areas (Table 5).

**Table 5:** Distribution of the scores of separate areas of SF-36 questionnaire before, after treatment, and 1 month after treatment (stages I, II, III), with respect to groups of subjects

	Group I (*n* = 32)	Group II (*n* = 30)	Group III (*n* = 30)	*p* value
	A (SD), score			
Physical activity				
Stage I	40.2 (20.3)	43.4 (18.4)	39.5 (16.7)	*F* = 0.357, *p* = 0.701
Stage II	54.3 (23.5)	52.0 (16.2)	43.7 (16.2)	*F* = 2.552, *p* = 0.084
Stage III	53.5 (25.1)	55.0 (17.2)	45.3 (16.3)	*F* = 2.021, *p* = 0.132
Restriction of activity due to physical problems				
Stage I	43.0 (41.8)	59.2 (41.3)	54.3 (42.8)	*χ*^2^ = 2.553, *p* = 0.279
Stage II	68.3 (35.3)	75.9 (32.4)	61.2 (38.1)	*χ*^2^ = 2.072, *p* = 0.355
Stage III	68.0 (37.7)	72.4 (34.9)	60.0 (38.1)	*χ*^2^ = 1.462, *p* = 0.481
Pain				
Stage I	58.7 (19.3)	52.5 (16.8)	58.5 (16.5)	*χ*^2^ = 2.219, *p* = 0.33
Stage II	43.4 (18.2)	42.8 (16.5)	54.4 (19.9)	*F* = 2.800, *p* = 0.056
Stage III	43.4 (21.3)	39.8 (17.2)	51.5 (22.7)	*F* = 2.492, *p* = 0.086
Overall health assessment				
Stage I	55.8 (15.7)	52.5 (11.4)	50.7 (15.7)	*F* = 0.999, *p* = 0.372
Stage II	51.2 (12.4)	51.2 (12.4)	49.3 (16.1)	*F* = 0.180, *p* = 0.835
Stage III	52.1 (13.3)	46.7 (12.1)	52.7 (16.2)	*F* = 1.611, *p* = 0.206
Energy levels/vitality				
Stage I	34.8 (6.4)	32.2 (8.3)	34.3 (11.4)	*χ*^2^ = 2.172, *p* = 0.338
Stage II	35.8 (7.4)	32.8 (8.4)	33.8 (10.8)	*χ*^2^ = 2.461, *p* = 0.292
Stage III	35.8 (6.0)	33.6 (7.9)	33.5 (10.5)	*χ*^2^ = 1.32, *p* = 0.517
Social function				
Stage I	41.5 (7.7)	41.9 (8.6)	41.5 (13.4)	χ^2^ = 0.478, *p* = 0.787
Stage II	42.7 (8.7)	42.5 (6.0)	41.5 (13.4)	*χ*^2^ = 1.291, *p* = 0.524
Stage III	42.2 (9.4)	41.4 (8.3)	43.7 (14.6)	*χ*^2^ = 0.19, *p* = 0.909
Restriction of activity due to emotional disorders				
Stage I	72.9 (39.2)	75.6 (36.0)	74.7 (34.1)	*χ*^2^ = 0.057, *p* = 0.972
Stage II	86.2 (30.5)	78.2 (34.8)	78.2 (32.5)	*χ*^2^ = 3.313, *p* = 0.191
Stage III	83.3 (33.1)	82.8 (32.9)	75.6 (34.9)	*χ*^2^ = 1.887, *p* = 0.389
Emotional state				
Stage I	38.1 (5.5)	38.3 (7.4)	38.0 (7.8)	*χ*^2^ = 0.143, *p* = 0.931
Stage II	41.7 (7.3)	39.8 (8.3)	38.8 (8.7)	*F* = 0.985, *p* = 0.378
Stage III	40.9 (5.7)	37.5 (7.7)	40.1 (9.1)	*χ*^2^ = 5.531, *p* = 0.063

Group I, provided with bath procedures; group II, provided with mud procedures; *A*, average; *SD*, standard deviation; *F*, by Fisher test; *χ*^2^, by Kruskal–Wallis test

On the basis of KOOS questionnaire, during stage I, there was no significant difference between the averages of any subscale of respondents of the intervention group (group I, group II) and the control group (group III). However, after treatment and after 1 month after treatment, average percentages of symptoms, stiffness, and pain of the intervention groups (group I, group II) were significantly better than those of the control group (group III) (Table 6).

**Table 6:** Distribution of data of KOOS questionnaire on the knee (before, after treatment, and after 1 month after treatment (stages I, II, III), with respect to groups of subjects

Subscales of KOOS questionnaire	Group I (*n* = 32)	Group II (*n* = 30)	Group III (*n* = 30)	*p* value
	A (SD), score			
Symptoms				
Stage I	51.9 (15.1)	50.5 (13.0)	51.2 (17.7)	*F* = 0.068, *p* = 0.934
Stage II	66.3 (15.3)^a^	61.5 (16.3)	53.9 (20.7)^a^	*F* = 3.881, ^a^*p* = 0.02
Stage III	67.2 (15.0)^a^	64.9 (16.8)	53.9 (20.7)^a^	*F* = 4.929, ^a^*p* = 0.012
Stiffness				
Stage I	55.4 (16.3)	53.8 (13.4)	49.1 (19.8)	*F* = 1.189, *p* = 0.309
Stage II	67.2 (13.9)^a^	64.5 (17.4)^b^	53.4 (21.6)^a,b^	*F* = 5.146, ^a^*p* = 0.009, ^b^*p* = 0.054
Stage III	70.4 (15.3)^a^	69.0 (16.5)^b^	53.6 (21.7)^a,b^	*F* = 8.112, ^a^*p* = 0.001, ^b^*p* = 0.004
Pain				
Stage I	53.1 (15.7)	48.3 (12.5)	47.1 (15.4)	*F* = 1.505, *p* = 0.228
Stage II	65.3 (16.4)^a^	60.3 (18.3)	51.0 (19.1)^a^	*F* = 5.023, ^a^*p* = 0.009
Stage III	65.2 (14.8)^a^	62.2 (18.1)	52.3 (19.8)^a^	*F* = 4.534, ^a^*p* = 0.014
Mobility, everyday life				
Stage I	28.8 (17.8)	25.5 (15.8)	35.2 (21.5)	*χ*^2^ = 3.548, *p* = 0.17
Stage II	40.6 (22.3)	42.0 (21.4)	39.8 (23.2)	*F* = 0.072, *p* = 0.930
Stage III	42.8 (21.2)	41.8 (21.8)	40.2 (21.6)	*F* = 0.119, *p* = 0.888
Mobility, sports and recreational activities				
Stage I	34.9 (16.6)	35.2 (20.6)	36.3 (15.8)	*F* = 0.048, *p* = 0.953
Stage II	44.3 (23.7)	46.1 (21.6)	39.2 (16.8)	*F* = 0.889, *p* = 0.415
Stage III	50.2 (22.4)	50.0 (22.2)	41.3 (18.1)	*F* = 1.742, *p* = 0.181

Group I, provided with bath procedures; group II, provided with mud procedures; group III, control; *A*, average; *SD*, standard deviation; *F*, by Fisher test; *χ*^2^, by Kruskal–Wallis test

^a,b^*p* < 0.05

The values of parameters of groups I and II, presented in Tables 2, 3, 4, 5, and 6, were not significantly different; therefore, we combined these groups in our prognostic analysis.

For variables with significantly different changes between groups I, II, and control group, we estimated optimal threshold values using ROC test (Table 7). On the basis of binary logistic regression analysis, we predicted chance relations for reaching values greater than optimal for group III subjects.

**Table 7:** Distribution of ROC test predicted values of changes of variables of clinical trials and changes of their characteristics after treatment and 1 month after treatment, according to groups of subjects

Associated criterion	AUC [95% CI], %	Sensitivity/specificity [95% CI], %	Group III/group I+II, *n* (%)	Group III OR [95% CI]
Walking speed after treatment ≤ 0.025 m/s	94,8 [85.4–97.1]	93.3 [77.9–99.2] 87.1 [76.1–94.3]	28 (93.3) 8 (12.9)	94.5 [18.789–475.285]
Walking speed 1 months after treatment ≤ 0.05 m/s	97.8 [92.4–99.8]	93.3 [77.9–99.2] 96.7 [88.7–99.6]	28 (93.3) 2 (3.3)	413.0 [55.287–3085.16]
5 sit downs/stand ups after treatment ≤ 1.92, s	72.3 [61.9–81.2]	69.0 [49.2–84.7] 77.4[65.0–87.1]	20 (69.0) 14 (22.6)	7.619 [2.841–20.434]
5 sit downs/stand 1 month after treatment ≤ 2.75 s	81.1 [71.5–88.6]	65.5 [45.7–82.1] 86.9 [75.8–94.2]	19 (65.5) 8 (13.1)	12.587 [4.33–36.596]
Left leg				
Flexion range after treatment ≤ 4.0°	82.0 [71.8–89.8]	56.7 [37.4–74.5] 91.8 [80.4–97.7]	17 (56.7) 4 (8.2)	14.712 [4.207–51.445]
Flexion range 1 month after treatment ≤ 5°	87.4 [77.9–93.8]	70.0 [50.6–85.3] 83.3 [69.8–92.5]	21 (70.0) 8 (16.7)	11.667 [3.926–34.666]
Right leg				
Flexion range after treatment ≤ 10.0°	82.7 [72.6–90.2]	90.0 [73.5–97.9] 60.0 [45.2–73.6]	27 (90.0) 20(40.0)	13.5 [3.606–50.545]
Flexion range 1 month after treatment ≤ 12.0°	88.0 [78.8–94.2]	93.3 [77.9–99.2] 63.3 [48.3–76.6]	28 (93.3) 18 (36.7)	24.111 [5.13–113.333]
Left leg				
Flexor strength after treatment ≤ 0 score	93.9 [86.1–98.0]	100 [88.4–100] 89.8 [77.8–96.6]	30 (100) 5 (10.2)	–
Flexor strength 1 month after treatment ≤ 0 score	93.8 [85.9–98.0]	100 [88.4–100] 87.5 [74.8–95.3]	30 (100) 6 (12.5)	–
Right leg				
Flexor strength after treatment ≤ 0 score	98.0 [91.9–99.8]	100 [88.4–100] 95.9 [86.0–99.5]	30 (100) 2 (4.1)	–
Flexor strength 1 month after treatment ≤ .0 score	94.9 [87.5–98.6]	100 [88.4–100] 89.8 [77.8–96.6]	30 (100) 5 (10.2)	–
Left leg				
Extensor strength after treatment ≤ 0 score	90.5 [81.8–95.9]	93.3 [77.9–99.2] 85.7 [72.8–94.1]	28 (23.3) 7 (14.3)	84.0 [16.252–434.166]
Extensor strength 1 month after treatment ≤ 0 score	89.5 [80.5–95.3]	93.3 [77.9–99.2] 83.3 [69.8–92.5]	28 (93.3) 8 (16.7)	70.0 [13.811–354.781]
Right leg				
Extensor strength after treatment ≤ 0 score	89.7 [80.6–95.5]	93.3 [77.9–99.2] 87.0 [73.7–95.1]	28 (93.3) 6 (13.0)	93.333 [17.542–496.59]
Extensor strength 1 month after treatment ≤ .0 score	87.0 [77.4–93.6]	93.3 [77.9–99.2] 78.7 [64.3–89.3]	28 (93.3) 10 (21.)	51.8 [10.505–255.437]

Group I, provided with bath procedures; group II, provided with mud procedures; group III, control; *OR*, odds ratio; *CI*, confidence interval; *AUC*, area under the ROC curve

On the basis of non-parametric Spearman analysis, it was found that categorized changes of all parameters presented in Table 7 correlated directly and significantly between themselves.

Distribution of threshold values and their characteristics of KOOS questionnaire’s changes of separate scales after treatment and changes 1 month after treatment is presented in Table 8. On the basis of non-parametric Spearman correlation analysis, we found that categorized changes of all parameters presented in Table 8 correlated directly and significantly between themselves.

**Table 8:** Distribution of ROC test predicted values of KOOS questionnaire’s on the knee changes of variables and changes of their characteristics after treatment and 1 month after treatment, according to groups of subjects

Associated criterion, score	AUC [95% CI], %	Sensitivity/specificity [95% CI], %	Group III/group I+II, *n* (%)	Group III OR [95% CI]
Symptoms after treatment ≤ 1.5	83.7 [74.5–92.8]	86.7 [79.7–93.6] 80.6 [72.6–88.7]	26 (86.7) 12 (19.4)	27.083 [7.942–92.363]
Symptoms 1 month after treatment ≤ 1.0	82.0 [72.7–91.4]	86.7 [79.7–93.6] 77.4 [68.9–86.0]	26 (86.7) 14 (22.6)	22.286 [6.65–74.686]
Stiffness after treatment ≤ 5.5	76.4 [66.2–86.6]	86.7 [79.7–93.6] 66.1 [56.5–75.8]	26 (86.7) 21 (33.9)	12.69 [3.912–41.165]
Stiffness 1 month after treatment ≤ 5.5	82.8 [73.6–92.1]	86.7 [79.7–93.6] 79.0 [70.7–87.4]	26 (86.7) 13 (21.0)	24.5 [7.252–82.769]
Pain after treatment ≤ 1.5	82.8 [73.6–92.1]	86.7 [79.7–93.6] 79.0 [70.7–87.4]	26 (86.7) 13 (21.0)	24.5 [7.252–82.769]
Pain 1 month after treatment ≤ 1.5	76.3 [65.7–86.9]	80.0 [71.8–88.2] 72.6 [63.5–81.7]	24 (80.0) 17 (27.4)	10.588 [3.689–30.389]
Mobility, everyday life after treatment ≤ 13.5	71.6 [60.8–82.3]	86.7 [79.7–93.6] 56.5 [46.3–66.6]	26 (86.7) 27 (43.5)	8.426 [2.625–27.047]
Mobility, everyday life 1 month after treatment ≤ 24.5	76.2 [65.5–87.0]	76.7 [68.0–85.3] 75.8 [67.1–84.6]	23 (76.7) 15 (24.2)	10.295 [3.688–28.736]
Mobility, sports and recreational activities after treatment ≤ 3.0	76.4 [66.2–86.6]	86.7 [79.7–93.6] 66.1 [79.7–93.6]	26 (86.7) 21 (33.9)	12.69 [3.912–41.165]
Mobility, sports and recreational activities 1 month after treatment ≤ 3.0	79.5 [69.3–89.7]	86.7 [79.7–93.6] 79.0 [70.7–87.4]	24 (80.0) 13 (21.0)	15.077 [5.102–44.557]

## Discussion

The positive effect of mineral water and mud therapy is associated with mechanical, thermal, and chemical effect; however, the mechanism of action remains a matter of debate. Kulisch et al. (2014) found the advantage of mineral water baths in reducing pain and improving functional state compared with freshwater therapy. Branco et al. (2016) provide data which shows that sulfur mineral baths (three 20-min baths a week for 10 weeks, at 37–39°) are superior in reducing pain and reducing stiffness in the long term compared with freshwater baths. A study which dealt with assessing the effectiveness of mud therapy carried out by Tefner et al. found that both mud packs and specially manufactured thermal packs equally significantly reduced pain, stiffness and improved functional state. Therefore, these authors concluded that the positive impact of mud packs was likely attributable to thermal effect, while chemical effect remained unproven (Tefner et al. 2013). Assessing stiffness and functional state, Güngen et al. (2012) found partial advantage of mud packs compared with thermal packs; meanwhile, assessing pain, both methods had equally significant positive effects. Systematic literature review and meta-analysis published by Antonelli et al. (2018) found statistically significant advantage of a real balneological intervention (thermal mineral water or mud packs), compared with placebo interventions (freshwater or thermal packs), assessing the quality of life of patients with knee joint OA. The results of our study coincide with the results of the aforementioned studies: treatment with mineral sodium chloride mineral baths, peat mud applications together with physiotherapy were more effective than only physiotherapeutic treatment. On the basis of ROC, optimal values were estimated for subjects of groups I and II, changes of which significantly differed from control group. On the basis of non-parametric Spearman correlation analysis, changes of presented anthropometric parameters of subjects of all groups and categorized changes of KOOS questionnaire’s data correlated between themselves after treatment and 1 month after treatment.

Despite increasing research showing the positive effect of natural factors, on the basis of knee joint OA treatment recommendations provided by international and national experts, the role of these treatment methods remains unclear. SPA therapy is mentioned in EULAR recommendations 2003 as one of the possible non-pharmacological knee joint OA treatment methods. However, updated EULAR recommendations 2013 do not mention SPA therapy (Fernandes et al. 2013; Jordan et al. 2003). OARSI recommends balneotherapy only for patients with knee joint plus other joint OA as well as with adjacent illnesses; meanwhile, there is a lack of evidence and recommendations for local knee joint OA (McAlindon et al. 2014).

## Limitation of the study

Limitations for this study may include evaluations of long-term (> 12 months) effects and quality of life. Moreover, the patients included in the study were all Kellgren and Lawrence grades 1 to 3. This is the patients who are willing to take an outpatient rehabilitation program. This is also an unavoidable limitation of our study and the results cannot be generalized to all knee OA patients, especially patients with particularly severe functional conditions.

## Conclusion

In the intervention groups of patients with OA of a knee joint, where natural factors were applied (mineral sodium chloride baths and peat mud applications), after treatment and after 1 month after treatment anthropometric data significantly improved, pain intensity and joint stiffness decreased, physical activity increased compared to the control group. Future randomized controlled studies are needed to confirm these results. Moreover, further studies involving a higher number of participants with a longer period of observation are encouraged in order to shed more light on this subject.
